# Reduced angiogenic gene expression in morbillivirus‐triggered oncolysis in a translational model for histiocytic sarcoma

**DOI:** 10.1111/jcmm.13023

**Published:** 2016-11-17

**Authors:** Vanessa Maria Pfankuche, Ingo Spitzbarth, Stefanie Lapp, Reiner Ulrich, Ulrich Deschl, Arno Kalkuhl, Wolfgang Baumgärtner, Christina Puff

**Affiliations:** ^1^ Department of PathologyUniversity of Veterinary MedicineHannoverGermany; ^2^ Department of Non‐Clinical Drug SafetyBoehringer Ingelheim Pharma GmbH&Co KGBiberach (Riß)Germany

**Keywords:** canine distemper virus, DH82, histiocytic sarcoma, macrophage polarization, microarray, oncolysis

## Abstract

Histiocytic sarcoma represents a rare malignant tumour with a short survival time, indicating the need of novel treatment strategies including oncolytic virotherapy. The underlying molecular mechanisms of viral oncolysis are largely unknown. As cancer in companion animals shares striking similarities with human counterparts, we chose a permanent canine histiocytic sarcoma cell line (DH82 cells) to identify global transcriptome changes following infection with canine distemper virus (CDV), a paramyxovirus closely related to human measles virus. Microarray analysis identified 3054 differentially expressed probe sets (DEPs), encoding for 892 up‐ and 869 down‐regulated unique canine genes, respectively, in DH82 cells persistently infected with the vaccine strain Onderstepoort of CDV (DH82‐Ond‐pi), compared to non‐infected DH82 cells. Up‐regulated genes were predominantly related to immune processes, as demonstrated by functional enrichment analysis. Moreover, there was substantial enrichment of genes characteristic for classically activated M1 and alternatively activated M2 macrophages in DH82‐Ond‐pi; however, significant polarization into either of both categories was lacking. ‘Angiogenesis’ was the dominant enriched functional term for the down‐regulated genes, highlighting decreased blood vessel generation as a potential mechanism of paramyxovirus‐induced oncolysis in DH82 cells. The anti‐angiogenic effect of infection was verified by immunohistochemistry, which revealed a lower blood vessel density in an *in vivo* mouse model, xenotransplanted with DH82‐Ond‐pi, compared to mice transplanted with non‐infected DH82 cells. Reduction in angiogenesis appears to be an important oncolytic mechanism of CDV in DH82 cells, suggesting that similar mechanisms might account for human histiocytic sarcoma and maybe other tumours in conjunction with measles virus.

## Introduction

Spontaneous tumours in companion animals are increasingly acknowledged as a suitable model for human cancer 1, in particular for rare neoplastic entities, and provide a unique chance for the development of novel therapeutics 2. Histiocytic sarcoma (HS) represents such a rare disease in human beings, predominantly affecting intestine, skin and soft tissue or occurring systemically 3, 4. The biological behaviour of HS and the underlying molecular mechanisms have been subject to detailed research infrequently, and an ideal treatment scheme is still lacking 5. Resembling the human counterpart in many aspects, the relatively higher prevalence of HS in dogs emphasizes the dog as an interesting translational animal model 6, 7, 8. Similar to human beings, canine histiocytic sarcoma may occur as a localized and a disseminated form 6. As sufficient therapies for HS are still lacking, HS is associated with a relatively short survival time, indicating the need for novel therapeutic approaches 9, 10, 11. Due to its infrequency, research on HS faces several limitations which might be overcome by permanent cell cultures to identify targets of therapeutic relevance. Human HS cell lines are so far lacking 12; however, a HS cell line derived from a canine patient (DH82 cells) is commercially available. The unique combination of histiocytic and malignant neoplastic features makes DH82 cells highly attractive for research 13.

One promising approach for cancer therapy is represented by the use of oncolytic viruses 14. Certain paramyxoviruses such as measles virus represent highly interesting candidates for the treatment of different human neoplasms with encouraging outcome in clinical trials 14. CDV, a paramyxovirus closely related to measles virus, has been demonstrated to be capable of infecting and inducing cytopathogenic effects in DH82 cells 15 leading to the hypothesis that CDV may exert oncolytic properties in this specific cell line, comparable to reported oncolytic effects of attenuated measles virus in other neoplasms 16, 17. Several principal biomechanisms may ultimately lead to oncolysis including direct virus‐mediated killing of cells, inhibition of proliferation, induction of apoptosis and inhibition of tumour angiogenesis 14. Moreover, modulation of the phenotype of tumour‐associated macrophages (TAM) might have pivotal effects on the fate of the tumour, as classically activated M1 macrophages and alternatively activated M2 macrophages have previously been shown to critically influence tumour regression and progression, respectively 18. CDV has previously been shown to be capable of influencing the polarization state of canine macrophages 19, and thus, the question arises whether CDV might also polarize neoplastic DH82 cells into a specific phenotype, thereby potentially exerting oncolytic properties.

If CDV indeed induces oncolytic effects in canine HS, one possible future therapeutic application might consist of infection of HS with attenuated strains of CDV. As an alternative future approach, DH82 cells persistently infected with CDV might be transplanted into canine patients with HS using these cells with an established and high infection rate as a permanent virus source. Interestingly, a DH82 cell line persistently infected with the vaccine strain Onderstepoort of CDV (CDV‐Ond) has previously been developed, which consistently displays a high infection rate of more than 85% infected cells and represents a suitable model to study the effects of a stable CDV infection upon this neoplastic cell line (DH82‐Ond‐pi) 20. DH82‐Ond‐pi may thus be regarded as a more suitable model to study CDV‐induced differences compared to acute infection, although the latter might clinically be more applicable. As possible oncolytic effects of CDV have not been addressed detailed so far, DH82‐Ond‐pi and non‐infected DH82 cells were compared in this study. The aim of this study was to (*i*) determine global transcriptome differences in non‐infected DH82 cells and DH82 cells persistently infected with CDV as a model for paramyxovirus‐induced transcriptional changes in HS with microarray technique. Moreover, in a hypothesis‐driven approach, (*ii*) we sought to determine the impact of CDV infection on the expression of either tumour‐suppressive M1 or tumour‐progressive M2 macrophage‐specific genes through the induction of a phenotype polarization in DH82 cells themselves, as DH82 cells unite a unique combination, that is macrophage origin and neoplastic transformation. Finally, based on the results of the global transcriptome analysis, (*iii*) we validated one potential mechanism of viral oncolysis in a mouse model, xenotransplanted with either persistently CDV‐infected or non‐infected DH82 cells. This study represents the first description of oncolytic effects of a persistent infection of CDV upon DH82 cells. Future studies will have to expand the presented findings with special regard to the question whether non‐persistent (*i.e*. acute) infection of canine HS will have similar oncolytic capacity.

## Materials and methods

### Cell culture

DH82 cells, received from the European Collection of Cell Cultures, and DH82 cells persistently infected with the CDV vaccine strain Onderstepoort (DH82‐Ond‐pi) were cultivated at 37°C in the presence of 5% CO_2_ in a loosely adherent monolayer in minimal essential medium (MEM) with Earle's salts (PAA, Cölbe, Germany), supplemented with 1% penicillin/streptomycin (PAA), 10% foetal calf serum (PAA) and 1% MEM non‐essential amino acids (Sigma‐Aldrich, Taufkirchen, Germany). DH82‐Ond‐pi were available within the department since their establishment as formerly described 20. Briefly, persistently infected DH82 cells were generated by infection of passage 104 of DH82 cells with the attenuated Onderstepoort strain of CDV (CDV‐Ond, kindly provided by Dr. Metzler, Institute of Virology, Veterinary Medical Faculty, University of Zürich, Switzerland) with a multiplicity of infection (MOI) of 1.0. To establish a stable population of persistently infected DH82 cells, which produces infectious virus particles in the culture supernatant, few surviving cells that did not undergo cytolysis were weekly passaged over a total number of 17 passages, which resulted in a stable persistently infected population.

### Immunofluorescence

To confirm successful CDV infection, immunofluorescence for detection of CDV nucleoprotein (CDV‐NP) was performed at day 1 after seeding of 30,000 cells/well [non‐infected DH82 cells (passage 10) and DH82‐Ond‐pi (passage 141)] in quadruplicates on 96 microwell plates (Nunc GmbH & Co. KG, Thermo Scientific, Langenselbold, Germany) according to a 2‐day protocol with minor variations 21. Cells were fixed with paraformaldehyde (4%). An anti‐CDV nucleoprotein (NP) antibody (D110; 1:100; monoclonal mouse anti‐CDV‐NP; kindly provided by Prof. A. Zurbriggen, University of Bern, Switzerland) served as a primary antibody and a Cy^™^ 3‐conjugated goat anti‐mouse IgG (H + L) antibody (1:100; Jackson ImmunoResearch Laboratories, Hamburg, Germany) as a secondary antibody. Bisbenzimide was used for nuclear counterstaining (Sigma‐Aldrich). CDV‐NP‐positive and CDV‐NP‐negative cells were counted in 10 randomly selected high power fields per well with a fluorescence microscope (Olympus IX70‐S8F2; Olympus Life Science Europe GmbH, Hamburg, Germany), and the median percentage of immunopositive cells was calculated.

### RNA isolation, hybridization, quality check and normalization of microarray data

RNA isolation was performed on four replicates of DH82‐Ond‐pi and non‐infected DH82 cells, respectively, 1 day after seeding of 750,000 cells in T25 flasks (Nunc GmbH & Co. KG, Thermo Scientific). Total RNA was extracted with TRIzol (Invitrogen^™^ GmbH, Darmstadt, Germany), digested with RNase‐free DNase (Qiagen GmbH, Hilden, Germany) and purified with a silica gel‐based membrane (RNeasy Mini Kit; Qiagen GmbH) according to the manufacturer's protocols 20, 22. The RNA integrity number (RIN) and quantity were controlled with an Agilent Bioanalyzer 2100 in combination with an Agilent 6000 RNA Nano Kit as described 23.

Two hundred nanogram of each of the eight RNA samples was amplified and biotin‐labelled employing the 3′IVT express kit (Affymetrix, Santa Clara, CA, USA) and hybridized to GeneChip Canine Genome 2.0 arrays (Affymetrix) in a rotating hybridization oven at 45°C for 16 hrs. Afterwards, the arrays were washed and stained with a solution containing R‐phycoerythrin–streptavidin employing the Affymetrix GeneChip Fluidics Station 450 24. Scanning was performed with an Affymetrix GeneChip Scanner 3000. Background adjustment, quantile normalization and probe set summarization were performed with the GC‐RMA algorithm (Bioconductor gcrma for R package, version 2.3; http://www.bioconductor.org/) 25. Principal component analysis (PCA) was conducted to visually assess homogeneity of the data sets, with the online tool ClustVis (http://biit.cs.ut.ee/clustvis/) 26. The data set was uploaded to ArrayExpress 27 and is available in the ArrayExpress database (http://www.ebi.ac.uk/arrayexpress) under accession number E‐MTAB‐3942.

### DEPs and probe set annotation

For detection of DEPs, a one‐factorial test with the Linear Models for Microarray Data (LIMMA) algorithm, implied in Babelomics 4.3 28, was used for pairwise comparison of DH82‐Ond‐pi and non‐infected DH82 cells. Multiple test correction was applied with a maximal false discovery rate of 5% (*q* ≤ 0.05), according to the method of Benjamini and Hochberg 28, 29. DEPs were filtered combining a highly stringent statistical significance filter (LIMMA, *q* ≤ 0.05) and a moderately stringent fold change (FC) filter (FC ≥ 2.0 or ≤ −2.0) 27. The FC was calculated as the ratio of the inverse‐transformed arithmetic means of the log_2_‐transformed expression values 24, 30. Down‐regulations are shown as negative reciprocal values 24. Probe sets were annotated with canine gene symbols and gene names according to the Affymetrix Annotation file (release 33; 29 October 2012). Differentially expressed genes (DEGs) were defined as probe sets with official canine gene symbol annotation. Selected non‐official gene symbols were added manually 24.

### Functional annotation based on the Gene Ontology database

Differentially expressed genes were assigned to functional terms in the Gene Ontology (GO) Biological Process category, applying WEB‐based GEne SeT AnaLysis Toolkit (WebGestalt; http://www.webgestalt.org/) 31, 32 and the Database for Annotation, Visualization and Integrated Discovery (DAVID) 33. For all analyses with WebGestalt, the Affymetrix Canine Genome 2.0 was used as reference. Based on the comparatively low amount of functional annotations of canine microarray data 24, the original canine gene lists were cross‐annotated into orthologous human gene symbols applying MADgene (http://cardioserve.nantes.inserm.fr/mad/madgene/) 34 when used as input data for the analyses applying DAVID with the human genome as reference background. For reasons of manageability, the number of enriched biological modules was limited to ≤ 10 30.

In addition, a more stringent FC filter of ≤ −5.0 and ≥ 5.0 was applied for some functional enrichment analyses, to consolidate large lists of DEGs to genes with the most prominent regulation.

### Manual generation of a gene list of macrophage phenotypes

Based on the histiocytic origin of DH82 cells and previous observations that CDV influences the polarization of canine macrophages 19, a previously generated literature‐based list of human and murine genes, specifically expressed by either M1 or M2 macrophages, was used as a basis to test whether CDV infection of DH82 cells induces a polarization of these cells into one of these categories 35, 36. This list was translated into canine orthologous gene symbols by employing MADgene 34 and the web‐based ‘information hyperlinked over proteins’ (ihop; http://www.ihop-net.org/UniPub/iHOP/) 37, resulting in a list of 65 canine genes for the M1 and 58 for the M2 category, respectively. For the M1 category, 59 genes (109 probe sets) were represented on the chip, whereas 55 genes (104 probe sets) were retrieved for the M2 category. The raw expression data for these genes were filtered employing SAS Enterprise Guide (SAS version 9.3; SAS Institute Inc, Cary, NC, USA) and compared between non‐infected and DH82‐Ond‐pi, employing multiple pairwise nonparametric Mann–Whitney *U*‐tests. Differential expression was defined by the combination of a statistical significance filter (Mann–Whitney *U*‐test; *P* ≤ 0.05) and an FC filter (FC ≥ 2.0 or ≤ −2.0).

### Histology and immunohistochemistry in a xenotransplantation mouse model

To test whether the observed transcriptome differences between infected and non‐infected DH82 cells indeed point to a capacity of CDV of inducing oncolysis *in vivo*, formalin‐fixed paraffin‐embedded (FFPE) material of a xenotransplantation *in vivo* mouse model, approved and authorized by the local authorities (Niedersächsisches Landesamt für Verbraucherschutz‐ und Lebensmittelsicherheit (LAVES), Oldenburg, Germany, permission number 33.9‐42502‐04‐08/1515), was used. All animal procedures were performed in accordance with the German regulations and legal requirements. A total number of 60 female severe combined immunodeficiency (SCID) mice (CB17/Icr‐*Prkdc* ^scid^/lcrlcoCrl) were subcutaneously xenotransplanted with non‐infected DH82 cells (group 1; *n* = 30) or DH82‐Ond‐pi (group 2; *n* = 30). Tumour growth was measured every 2–3 days. For histological and immunohistochemical investigations in this study, necropsy and sample collection were performed on day 7, 14, 21, 35 and 77 after xenotransplantation (days post transplantation (dpt); *n* = 6 animals per time point and group). However, due to histologically determined complete regression of DH82‐Ond‐pi neoplasms in four of six animals at 35 dpt and six of six animals at 77 dpt, detailed histological and immunohistochemical analyses were only performed at 7, 14 and 21 dpt.

Haematoxylin–eosin‐stained histological sections of tumours were morphometrically analysed to determine total tumour area and quantify areas of necrosis. The percentage of necrotic tumour areas, characterized by hypereosinophilia, loss of cell borders, accumulation of cellular debris, karyopyknosis, karyorrhexis and karyolysis, was determined with the analySIS 3.1 software (Soft Imaging System, Münster, Germany).

Immunohistochemistry with the avidin–biotin–peroxidase complex method was performed with a panel of polyclonal rabbit anti‐human antibodies. To investigate the intratumoural vascularization, a CD31/PECAM antibody (1:50; AP15436PU‐M, Acris Antibodies GmbH, Herford, Germany) was applied as described 22. To quantify potential apoptotic processes, immunohistochemistry for cleaved caspase‐3 was performed (1:200; Asp175, Cell Signaling Technology Inc., Danvers, MA, USA). The proliferation of tumours was assessed by immunohistochemistry for Ki67 (1:100; Clone MIB‐1, DakoCytomation GmbH, Hamburg, Germany). For all immunohistochemical procedures, FFPE sections were deparaffinized and rehydrated through graded alcohols. 0.5% H_2_O_2_ in ethanol served as suppressor for endogenous peroxidase activity. Sections were pre‐treated with citric buffer followed by blocking (goat serum 1:5) and addition of a biotinylated goat anti‐rabbit secondary antibody (1:200). 3,3`‐diaminobenzidine‐tetrahydrochloride served as chromogen followed by slight counterstaining with Mayer's hemalaun (Carl Roth GmbH, Karlsruhe, Germany).

Immunopositivity for cleaved caspase‐3 and Ki67 was evaluated morphometrically and was given as the percentage of immunopositive area related to total tumour area. As tumour cells of HS have previously been reported to be CD31‐immunopositive 11, 38, 39, only CD31‐positive structures containing a definable lumen, interpreted as blood vessels, were counted in the whole tumour area. The intratumoural blood vessel density was calculated per square mm. Statistical analysis for all immunohistochemical data was performed with multiple Mann–Whitney *U*‐tests. *P*‐values below 0.05 were considered significant.

## Results

### Morphology and immunofluorescence

**Figure 1:**
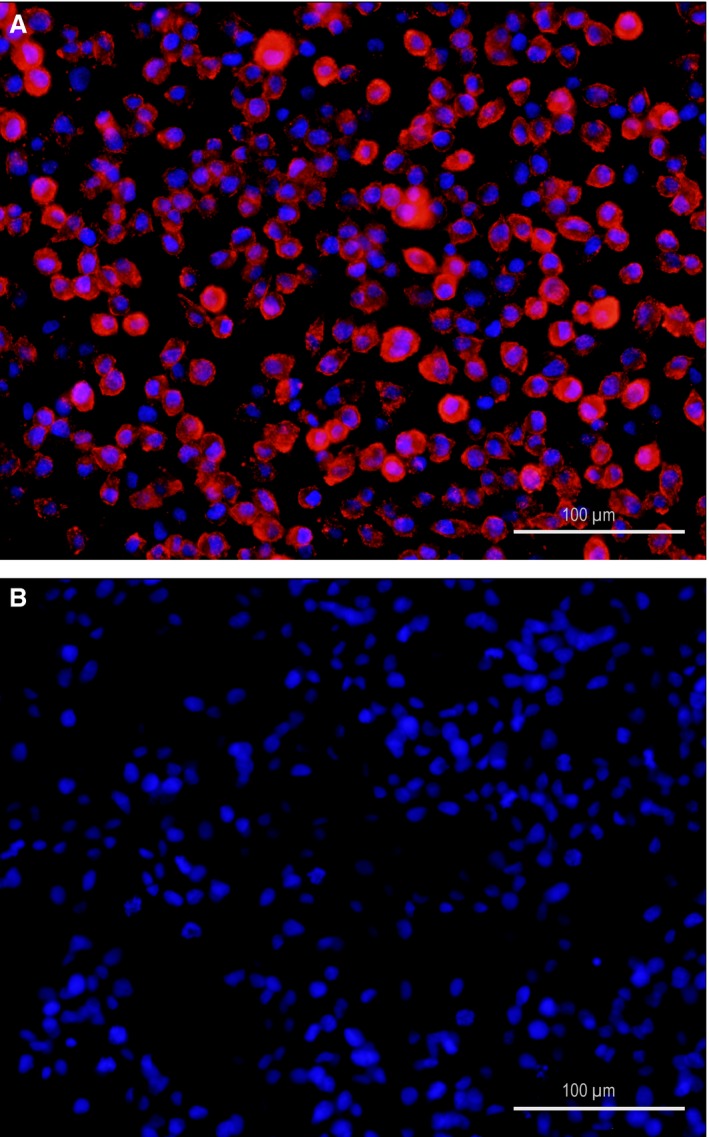
DH82 cells persistently infected with the Onderstepoort strain of canine distemper virus (**A**) revealed a strong cytoplasmic immunopositivity for the nucleoprotein of canine distemper virus (CDV‐NP, red), whereas immunopositivity for CDV‐NP is lacking in non‐infected DH82 cells (**B**). Immunofluorescence for CDV‐NP; primary antibody: monoclonal mouse anti‐CDV‐NP (D110); secondary antibody: Cy^™^ 3‐conjugated goat anti‐mouse IgG. Bisbenzimide for nuclear counterstaining (blue). Scale bars = 100 μm.

Irrespective of their infection status and consistent with the literature 13, 20, 40, DH82 cells were plastic‐adherent and exhibited a typical macrophage‐like morphology ranging from 25 to 55 μm in diameter with round to oval, eccentrically located nuclei, with occasional multinucleated cells with cytoplasmic vacuoles. CDV nucleoprotein (CDV‐NP) was demonstrated in none of the controls, whereas a median percentage of 94.91% (min: 92.99%; max: 98.36%) of persistently CDV (strain Onderstepoort)‐infected DH82 cells (DH82‐Ond‐pi) revealed a strong cytoplasmic immunopositivity for CDV‐NP (Fig. 1).

### Persistent CDV infection of DH82 cells induces marked changes in the transcriptome level

**Figure 2:**
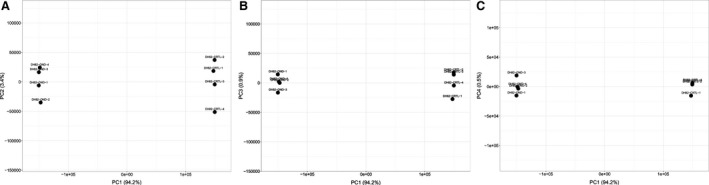
Principal component analysis of the microarray data of the four biological replicates of DH82 cells, persistently infected with the Onderstepoort strain of canine distemper virus (DH82‐OND) and non‐infected DH82 cells (DH82‐CTRL) as obtained by applying the online tool ClustVis (http://biit.cs.ut.ee/clustvis/) 26. Displayed are the combinations of the principal component (PC) one and two (**A**), PC one and three (**B**) and PC one and four (**C**). Only one major PC was identified, which explained 94.2% of the observed variance in the transcriptome data (PC1), which resulted in markedly isolated clusters of infected and non‐infected DH82 cells, respectively. The remaining components (PC2, PC3 and PC4) only explained relatively low percentages (0.5–3.4%) of the observed variance, thus indicating that PC1 most probably represents the infection status of the cells.

Principal component analysis (PCA) of the normalized data set revealed a clear distinction between the global expression data from the four replicates of infected and non‐infected DH82 cells with markedly isolated clusters (Fig. 2). One major principal component was identified, thus most likely representing the persistent infection of the cells, implying that the infection status and not other factors such as differences in the passage number explains the observed variances. This potent distinction between infected and non‐infected DH82 cells was mirrored by the relatively high number of 3054 probe sets (of 43035 probe sets displayed on the array) that were classified as differentially regulated (DEPs, Table 1). DEPs compartmentalized in a highly similar number of either up‐ (1504 DEPs) or down‐regulated probe sets (1550 DEPs) corresponding to 892 up‐ and 869 down‐regulated unique canine genes (DEGs; Table 1).

**Table 1:** Summary of differentially expressed probe sets in DH82 cells persistently infected with the Onderstepoort strain of canine distemper virus compared to non‐infected DH82 cells. Genes and enriched biological modules, retrieved by functional enrichment analysis with different annotation tools

Number of differentially expressed probe sets	Direction of regulation	Number of differentially expressed genes^a^	Enriched biological modules (WebGestalt)	Enriched biological modules (DAVID)^b^
3054	Up: 1504	Up: 892	Immune response‐activating signal transduction (*P* < 0.01); Activation of immune response (*P* < 0.01); Immune response‐regulating signalling pathway (*P* < 0.01); Positive regulation of immune response (*P* < 0.01); Response to other organism (*P* < 0.01); Regulation of immune response (*P* < 0.01); Positive regulation of immune system process (*P* < 0.01); Regulation of immune system process (*P* < 0.01); Immune response (*P* < 0.01); Immune system process (*P* < 0.01)	Activation of innate immune response (ES^c^: 3.029); Cell migration (ES: 2.531); Leucocyte proliferation (ES: 1.951); Positive regulation of programmed cell death (ES: 1.793); Positive regulation of leucocyte activation (ES: 1.718); Regulation of leucocyte proliferation (ES: 1.700); Blood coagulation (ES: 1.405)
	Down: 1550	Down: 869	Blood vessel morphogenesis (*P* = 0.027); Positive regulation of cell migration (*P* = 0.027); Positive regulation of cell motility (*P* = 0.027); Cardiovascular system development (*P* = 0.027); Positive regulation of cellular component movement (*P* = 0.027); Circulatory system development (*P* = 0.027); Regulation of cell adhesion (*P* = 0.027); Positive regulation of locomotion (*P* = 0.027); Localization of cell (*P* = 0.031); Biological adhesion (0.031)	Blood vessel development (ES: 2.174); Protein amino acid glycosylation (ES: 1.933); Organic acid metabolic process (ES: 1.907); Regulation of neurological system process (ES: 1.568); Regulation of transferase activity (ES: 1.300); Blood coagulation (ES: 1.294); Nucleobase, nucleoside and nucleotide metabolic process (ES: 1.234); Antigen receptor‐mediated signalling pathway (ES: 1.232); Leucocyte proliferation (ES: 1.217)

a Genes are defined as probe sets with unique canine gene symbol annotation.

b Biological modules retrieved by functional enrichment analysis of orthologous human gene symbols.

c Enrichment score.

The used Affymetrix Canine Genome 2.0 Array also includes seven probe sets targeting different RNAs of the CDV genome 24. These probe sets are annotated with genes encoding for six CDV proteins, the phosphoprotein (CDV P), the nucleocapsid protein (CDV N), the haemagglutinin protein (CDV H), the matrix protein (CDV M), the fusion protein (CDV F) and the large polymerase protein (CDV L) 24. In fact, all of the seven probe sets showed marked differential regulation as compared to non‐infected controls, with enormously high FCs ranging from 15,224.3 to 1336.3, thus underlining the infection status of the cells and demonstrating that persistent infection in fact leads to transcription of genes encoding for the represented proteins of the virus.

The top hits of up‐regulated canine DEGs (Table 2) included *DDX60* (*DEAD (Asp‐Glu‐Ala‐Asp) box polypeptide 60*), *DLA‐79* (*MHC class Ib*) and *CXCR7* with FCs ranging from 2198.9 to 196.6.

**Table 2:** The top 10 of up‐ and down‐regulated official canine gene symbols of the global transcriptome analysis of DH82 cells persistently infected with canine distemper virus compared to non‐infected DH82 cells

Gene symbol	Gene title	Fold change
Up‐regulated		
DDX60	DEAD (Asp‐Glu‐Ala‐Asp) box polypeptide 60	2198.944
DLA‐79	MHC class Ib	458.861
CXCR7	chemokine (C‐X‐C motif) receptor 7	196.584
F13A1	coagulation factor XIII, A1 polypeptide	189.515
LOC100685890	CMRF35‐like molecule 1‐like	156.689
CCR5	chemokine (C‐C motif) receptor 5 (gene/pseudogene)	150.471
TRIM22	tripartite motif containing 22	139.552
LOC100686473	uncharacterized LOC100686473	138.308
GPR34	G protein‐coupled receptor 34	130.582
ENPEP	glutamyl aminopeptidase (aminopeptidase A)	122.955
Down‐regulated		
SERPINB2	serpin peptidase inhibitor, clade B (ovalbumin), member 2	−1663.774
TPM2	tropomyosin 2 (*β*)	−777.085
SCIN	adseverin‐like	−677.097
VEGFB	vascular endothelial growth factor B	−593.197
THBS2	thrombospondin 2	−451.295
COL4A1	collagen, type IV, *α*‐1	−364.238
DMD	dystrophin	−326.126
S100P	S100 calcium‐binding protein P	−304.859
LOC608476	fatty acid‐binding protein, adipocyte‐like	−270.291
GSTA3	glutathione S‐transferase *α*‐3	−238.561

The ten top hits of down‐regulated DEGs (Table 2) comprised genes such as *SERPINB2* (*serpin peptidase inhibitor, clade B (ovalbumin), member 2*), *TPM2* (*tropomyosin 2 (β)*), *VEGFB* (*vascular endothelial growth factor B*), *THBS2* (*thrombospondin 2*), *SCIN* (*adseverin‐like*), *S100P* (*S100 calcium‐binding protein P*) *LOC608476* (*fatty acid‐binding protein, adipocyte‐like*), *GSTA3* (*glutathione S‐transferase α‐3*), *TCEAL1* (*transcription elongation factor A (SII)‐like 1*) and *COL4A1* (*collagen, type IV, α‐1*) with FCs ranging from −1663.8 to −208.9.

### Functional annotation of DEGs reveals multiple biological processes, which are affected by CDV infection of DH82 cells

The retrieved gene ontology (GO) terms of the biological process category are summarized in Table 1. Up‐regulated genes were predominantly related to positive regulation of immune processes. Moreover, the term ‘response to other organism’ was retrieved, thus most probably reflecting genes, which were induced by CDV infection. Besides biological processes associated with the innate immune system, cell migration, apoptosis and blood coagulation, GO terms such as ‘activation of innate immune response’ and terms involved in leucocyte activation and proliferation were retrieved. Interestingly, both independent functional enrichment analysis tools identified GO terms related to blood vessel development and morphogenesis as being markedly enriched for the down‐regulated genes (Table 1). Here, the term ‘blood vessel development’ was the most enriched biological module including genes such as *collagen type I, α‐1 (COL1A1)*,* endothelin 1 (EDN1)*,* integrin α‐7 (ITGA7)*,* cysteine‐rich angiogenic inducer 61 (CYR61)* and *chemokine (C‐X‐C motif) receptor 4 (CXCR4)*. Similarly, WebGestalt identified the term ‘blood vessel morphogenesis’ as being functionally enriched (Table 1). This term comprised 30 genes (Table 3) with *vascular endothelial growth factor B (VEGFB)* being the member with the highest absolute FC of −593.2, followed by *thrombospondin 2* (*THBS2*; −451.3). Besides, WebGestalt identified enriched GO terms related to cell migration, movement, localization and adhesion for the down‐regulated DEGs (Table 1). Different GO terms involved in cellular metabolic processes were retrieved as output with DAVID (Table 1).

**Table 3:** Members of the term ‘blood vessel morphogenesis’, identified as functionally enriched for the down‐regulated genes in DH82 cells persistently infected with canine distemper virus by WebGestalt

Gene symbol (*Canis familiaris*)	Gene title (*Canis familiaris*)	Fold change
VEGFB	vascular endothelial growth factor B	−593.197
THBS2	thrombospondin 2	−451.295
LOC476202	connective tissue growth factor‐like	−95.736
EDN1	endothelin 1	−47.795
F3	coagulation factor III (thromboplastin, tissue factor)	−32.838
ITGA7	integrin, *α*‐7	−30.731
CYR61	cysteine‐rich, angiogenic inducer, 61	−25.432
HPGD	hydroxyprostaglandin dehydrogenase 15‐(NAD)	−18.954
FGFR2	fibroblast growth factor receptor 2	−17.441
CXCR4	chemokine (C‐X‐C motif) receptor 4	−13.485
SERPINE1	serpin peptidase inhibitor, clade E (nexin, plasminogen activator inhibitor type 1), member 1	−13.116
LOC100856661	prostacyclin synthase‐like	−8.263
SERPINF1	serpin peptidase inhibitor, clade F (*α*‐2‐antiplasmin, pigment epithelium derived factor), member 1	−7.587
SRPX2	sushi repeat‐containing protein, X‐linked 2	−7.472
HYAL1	hyaluronoglucosaminidase 1	−7.376
COL4A2	collagen, type IV, *α*‐2	−5.747
CXCL12	chemokine (C‐X‐C motif) ligand 12	−3.683
NTRK2	neurotrophic tyrosine kinase, receptor, type 2	−3.568
CAV1	caveolin 1, caveolae protein, 22 kD	−3.382
GJC1	gap junction protein, γ 1, 45 kD	−3.325
RASA1	RAS p21 protein activator (GTPase‐activating protein) 1	−3.309
EGFL7	EGF‐like domain, multiple 7	−3.294
PTK2	PTK2 protein tyrosine kinase 2	−3.226
BMP4	bone morphogenetic protein 4	−3.050
ITGB1	integrin, *β*‐1 (fibronectin receptor, *β*‐polypeptide, antigen CD29 includes MDF2, MSK12)	−2.548
LEF1	lymphoid enhancer‐binding factor 1	−2.292
POFUT1	protein O‐fucosyltransferase 1	−2.213
ZFPM2	zinc finger protein, multitype 2	−2.135
FOXM1	forkhead box M1	−2.072

A second confirmatory approach with a more stringent FC filter of an absolute FC value of 5.0 resulted in a list of 186 up‐ and 218 down‐regulated DEGs, which served as input data for WebGestalt (data not shown). Interestingly, and similar to the functional analyses of the DEGs with a more moderate FC filter, ‘regulation of angiogenesis’ was retrieved as enriched functional term (*P* < 0.001). The most significant category for the up‐regulated canine genes was ‘defence response to virus’ and ‘activation of immune response’, both with *DDX60* as the gene with the highest FC (2198.9).

### Unsupervised hierarchical clustering analysis illustrates infection‐induced transcriptional changes in biological processes related to the immune response and angiogenesis

**Figure 3:**
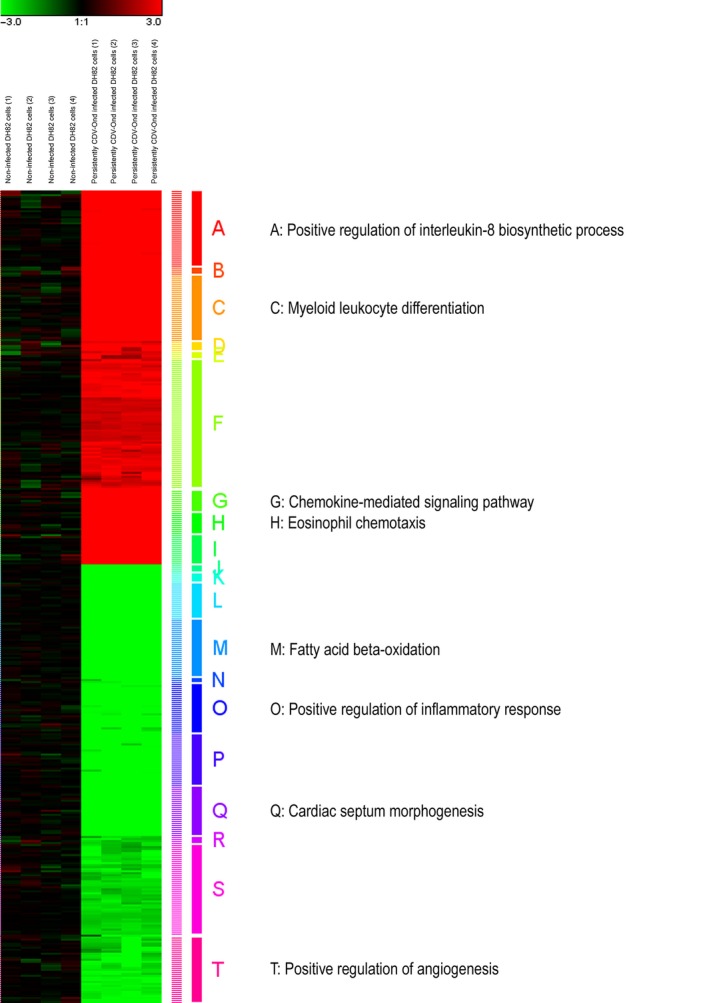
A highly stringent fold change filter (≥5.0 and ≤−5.0, respectively) was used for unsupervised hierarchical clustering of 404 canine differentially expressed genes in quadruplicates of non‐infected DH82 cells and DH82 cells persistently infected with the Onderstepoort strain of canine distemper virus (CDV‐Ond). Hierarchical clustering (Euclidean distance, complete linkage) of the logarithmized individual FCs 24 graphically illustrates the relatively similar proportions of up‐ (red colour) and down‐regulated genes (green colour) and resulted in 20 separate clusters of genes with similar expression patterns. The gene members of the clusters were used as input data for functional enrichment analysis with WebGestalt, resulting in eight clusters with significant enrichment in the biological process category of the Gene Ontology (GO) database (*P* < 0.05). The significant retrieved enriched GO terms are depicted.

The more stringent FC filter (≥ 5.0 or ≤ −5.0) was used for unsupervised hierarchical clustering of the 404 canine DEGs. Hierarchical clustering (Euclidean distance, complete linkage) of the logarithmized individual FCs graphically illustrated the relatively similar proportions of up‐ and down‐regulated genes (Fig. 3) and resulted in 20 separate clusters of genes with similar expression patterns 24. The cluster members were used as input data for functional enrichment analysis with WebGestalt (Fig. 3). For eight clusters, GO terms reached significance (*P* ≤ 0.05). Whereas ‘positive regulation of interleukin‐8 biosynthetic process’ (*P* = 0.006), ‘myeloid leucocyte differentiation’ (*P* = 0.008), ‘chemokine‐mediated signalling pathway’ (*P* = 0.003) and ‘eosinophil chemotaxis’ (*P* = 0.001) was retrieved for the clusters of up‐regulated genes, ‘fatty acid *β*‐oxidation’ (*P* = 0.027), ‘positive regulation of inflammatory response’ (*P* = 0.029), ‘cardiac septum morphogenesis’ (*P* = 0.008) and ‘positive regulation of angiogenesis’ (*P* = 0.008) reached significance for the down‐regulated gene clusters.

### CDV infection of DH82 cells induces transcriptional changes in genes specific for distinct macrophage phenotypes

**Figure 4:**
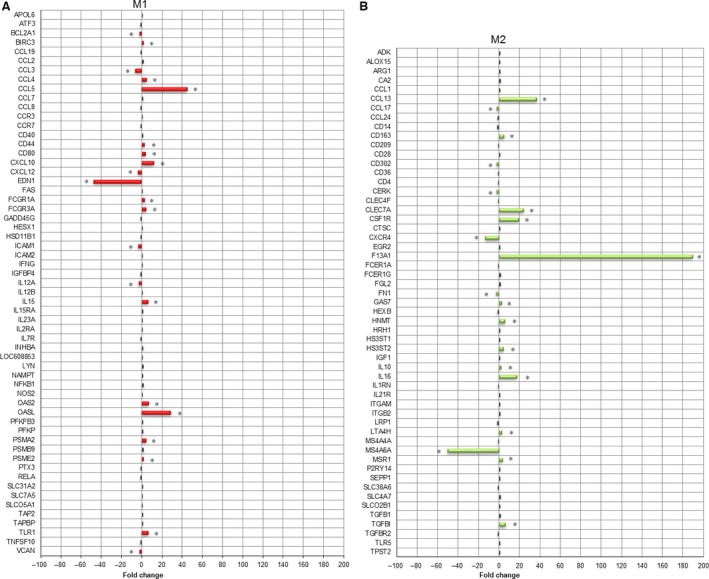
A previously generated literature‐based list of manually selected human and murine genes, specifically expressed by either M1 or M2 macrophages, was used as a basis to test whether CDV infection of DH82 cells induces a polarization of canine macrophages in one of these categories. Gene expression data for the M1 and M2 category were compared between non‐infected and persistently CDV‐Ond‐infected DH82 cells, employing multiple pairwise nonparametric Mann–Whitney *U*‐tests. Differential expression was defined by the combination of a statistical significance filter (Mann–Whitney *U*‐test; *P* ≤ 0.05) and a fold change filter (FC ≥ 2.0 or ≤ −2.0). A total of 21 of the 59 unique canine gene symbols for the M1 category were differentially regulated (**A**; red and marked by asterisks), whereas 19 of 55 genes exhibited differential regulation for the M2 category (**B**; green and marked by asterisks), respectively.

Classical (M1) and alternative (M2) activation of macrophages is a critical determinant for the fate of malignancies. Although generally applying to TAM, the unique histogenesis of HS raises the question whether persistent infection might similarly induce a phenotypical polarization in neoplastic DH82 cells themselves, thereby potentially exerting oncolytic properties. Thus, besides the global analysis, the data set was analysed for changes in the expression of literature‐based genes specifically expressed by M1‐ or M2‐polarized macrophages 35, 36. Persistent CDV infection caused a significant difference (Mann–Whitney *U*‐test *P* ≤ 0.05 and FC ≤ −2.0 or ≥ 2.0) in the expression of 21 of 59 unique canine gene symbols for the M1 category (14 up‐regulated; 7 down‐regulated), whereas 19 of 55 genes exhibited differential regulation for the M2 category (13 up‐regulated; 6 down‐regulated), respectively (Fig. 4). For the M1 category, *CCL5*,* OASL, CXCL10*,* OAS2* and *TLR1* were identified as the significantly up‐regulated genes with the highest FCs ranging from 44.9 to 6.6. The significantly up‐regulated genes of the M2 category comprised genes such as *F13A1*,* CCL13*,* CLEC7A*,* CSFR1* and *IL‐16* with FCs ranging from 189.5 to 17.4.

Fisher's exact test for significant enrichment in either of the both categories failed to reach significance (*P* > 0.05).

### Xenotransplanted DH82 cells persistently infected with CDV display reduced vascularity compared to non‐infected controls in an *in vivo* mouse model

Based on the results of transcriptome investigations, we hypothesized that persistent CDV infection might lead to reduced vascularity in xenotransplants of DH82 cells in SCID mice, thus supporting decreased angiogenesis as a potential mechanism of viral oncolysis in this model.

**Figure 5:**
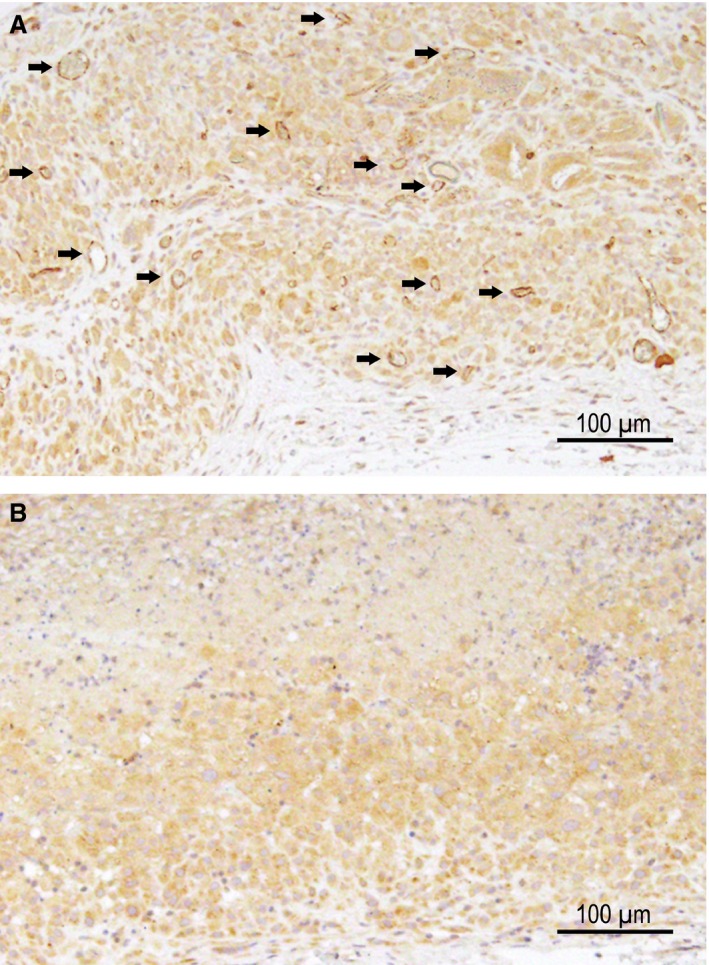
Immunohistochemistry for CD31 in a mouse model, xenotransplanted with non‐infected DH82 cells (**A**) and DH82 cells persistently infected with the Onderstepoort strain of canine distemper virus (CDV‐Ond; (**B**) 7 days after transplantation. Arrows highlight several immunopositive luminal structures (intratumoural blood vessels) in non‐infected DH82 cell transplants (**A**). In persistently CDV‐Ond‐infected DH82 cell transplants, the tumour lacks CD31‐immunopositive structures (**B**). Note that tumour cells stain slightly positive for CD31 in both infected and non‐infected transplants. Scale bars = 100 μm. Avidin–biotin–peroxidase method; chromogen = 3,3′‐diaminobenzidine.

**Figure 6:**
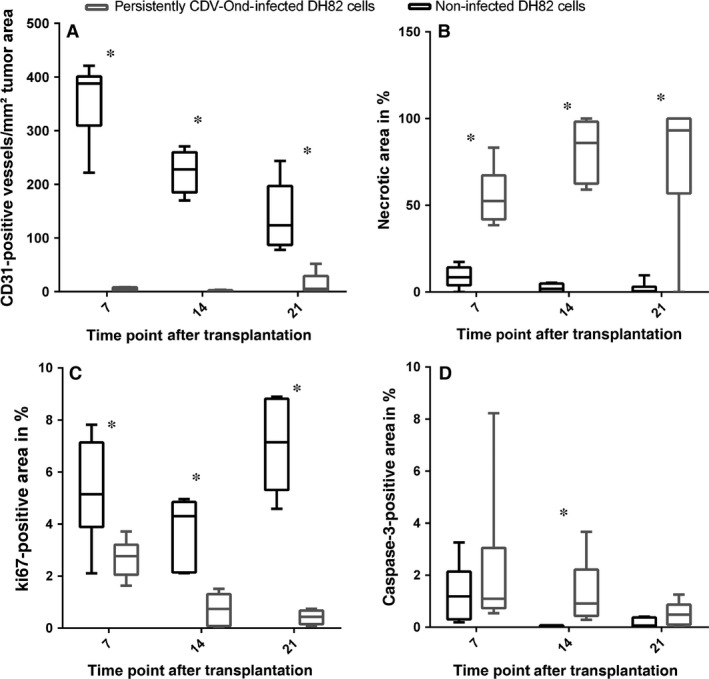
Box and whisker plots illustrating the differences between the number of CD31‐immunopositive luminal structures (**A**), necrotic area (**B**), Ki67‐ (**C**) and cleaved caspase‐3‐ (**D**) immunopositive area in a mouse model, xenotransplanted with DH82 cells and DH82 cells persistently infected with canine distemper virus (*n* = 6 per group and time point), respectively. (**A**) Intratumoural blood vessel density is significantly higher in non‐infected DH82 cell transplants (black) than in infected xenotransplants (grey) on day 7, 14 and 21 after transplantation. Statistical significance is indicated with an asterisk (*P* ≤ 0.05; multiple Mann–Whitney *U*‐tests). (**B**) DH82‐Ond‐pi tumours displayed significantly and markedly larger areas of necrosis than non‐infected controls at all histologically investigated time points (day 7, 14 and 21; *P* < 0.05; multiple Mann–Whitney *U*‐tests). (**C**) Tumour cell proliferation as determined by immunohistochemistry for Ki67 was significantly higher in non‐infected transplants compared to DH82‐Ond‐pi tumours at all investigated time points (day 7, 14 and 21; *P* < 0.05; multiple Mann–Whitney *U*‐tests). (**D**) At day 14, cleaved caspase‐3 antigen‐positive area was significantly larger in DH82‐Ond‐pi xenografts compared to non‐infected control tumours (*P* < 0.05; multiple Mann–Whitney *U*‐tests).

Tumour sections of non‐infected and DH82‐Ond‐pi xenotransplants on day 7, 14 and 21 following transplantation were evaluated. Pairwise comparison revealed a significantly higher intratumoural blood vessel density on day 7 (*P* = 0.005), 14 (*P* = 0.005) and 21 (*P* = 0.012) in tumours composed of non‐infected DH82 cells compared to DH82‐Ond‐pi (Figs 5 and 6A; Table 4). The comparison of vessel density between different time points of DH82‐Ond‐pi revealed no significant differences (*P* > 0.05).

**Table 4:** Median, minimum (min) and maximum (max) of the intratumoural blood vessel density at different time points after transplantation are displayed tabularly

CD31‐positive blood vessels/mm²	Time point after transplantation [d]		
	7	14	21
Persistently CDV‐Ond‐infected DH82 cell transplants			
Median	3.36	1.54	5.29
Min	0.798	0	0
Max	8.24	3.43	52
Non‐infected DH82 cell transplants			
Median	388	228	124
Min	222	170	77.8
Max	421	271	244

### Persistent infection with CDV is associated with tumour regression accompanied by increased necrosis in a DH82 xenotransplantation mouse model

**Figure 7:**
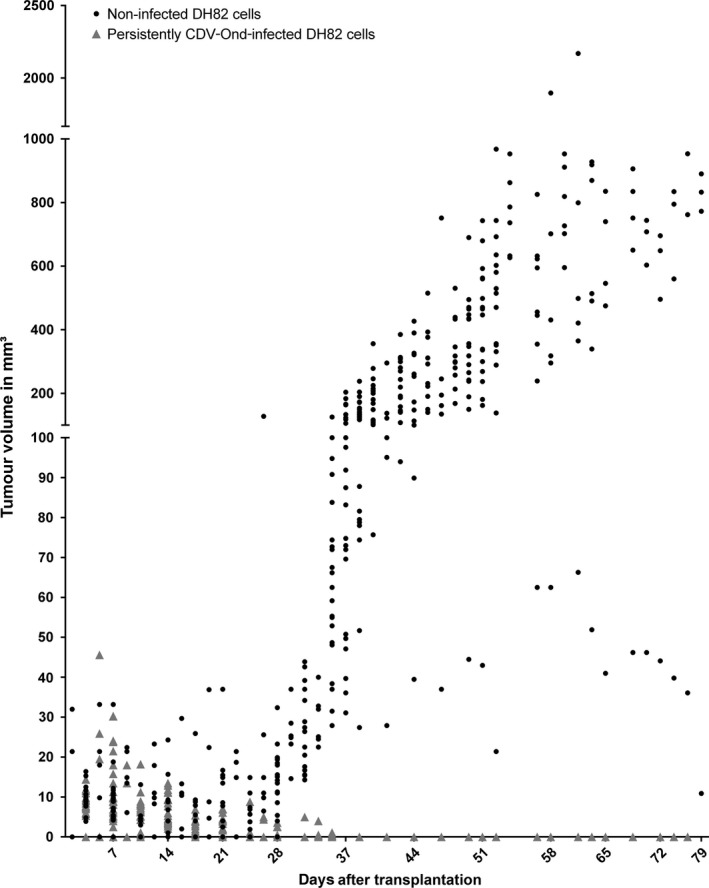
The figure shows a comparison of DH82 and persistently CDV‐infected DH82 cell (DH82‐Ond‐pi) xenotransplant volumes as determined clinically. DH82‐Ond‐pi neoplasms revealed a constant regression, starting at 14 days after transplantation (dpt), in which 35 dpt represented the last time point with clinically identifiable, small DH82‐Ond‐pi tumours (max. 4 mm³) in few animals (5/12). In contrast, DH82 xenografts exhibited a constant increase in tumour volume starting at 24 dpt. Except at day 31 after transplantation, DH82‐Ond‐pi tumours were significantly smaller than DH82 xenografts after 24 dpt (*P* < 0.05).

Starting at day 14 after transplantation, tumour sizes of DH82‐Ond‐pi transplants revealed a constant regression over the observed time period (Fig. 7). Overall, DH82‐Ond‐pi tumours displayed significantly and markedly larger areas of necrosis than non‐infected controls at all histologically investigated time points (day 7, 14 and 21; Fig. 6B; *P* < 0.05).

The percentage of cleaved caspase‐3 antigen‐positive area displayed no significant differences in both infected and non‐infected tumours at 7 and 21 days after transplantation. However, at day 14, cleaved caspase‐3 antigen‐positive area was significantly larger in DH82‐Ond‐pi xenografts compared to non‐infected control tumours (Fig. 6D; *P* < 0.05).

Tumour cell proliferation as determined by immunohistochemistry for Ki67 was significantly higher in non‐infected transplants compared to DH82‐Ond‐pi transplants at all investigated time points (day 7, 14 and 21; Fig. 6C; *P* < 0.05).

The average mitotic rate was four per high power field in both infected and non‐infected xenografts at day 7 after transplantation. Corresponding to the results of Ki67 immunostaining, at 21 days after transplantation, 0–1 mitotic figure per high power field was found in DH82‐Ond‐pi tumours, whereas non‐infected DH82 neoplasms showed up to 20 mitoses per high power field.

## Discussion

Viral oncolysis has evolved as a promising treatment strategy for certain forms of malignancies. We chose a microarray approach to identify transcriptional changes in an infected canine HS cell line as a translational model for the devastating HS in human beings. The present study clearly demonstrates that persistent CDV infection causes dramatical differences in the transcriptome of canine DH82 cells. Moreover, this is the first study demonstrating oncolytic effects of a persistent CDV infection in a canine HS cell line. To set up a basis for future studies on acute infection models and clinical applications, we sought to focus on a cell line with an established and persistent high infection rate.

Functional annotation of the up‐regulated genes in DH82‐Ond‐pi predominantly yielded GO terms related to activation of immune processes. These findings demonstrate that persistent CDV infection of DH82 cells induces marked up‐regulation of immune response‐related genes, which may be attributed to the unique combination of both histiocytic (*i.e*. hematopoietic) origin and malignant neoplastic behaviour of DH82 cells 13. Independent previous and ongoing studies have shown that non‐infected and persistently CDV‐infected DH82 cells do not show differences in their growth rate and cell viability *in vitro* as determined by trypan blue staining 20. Previous studies on non‐infected cells have, however, shown that DH82 cells may show diminished expression of immune‐related surface molecules such as CD11c^−^, CD14^−^, CD18^−^, CD45^−^ and CD80 during long‐term passaging 13. Probe sets encoding for these molecules were either up‐ or not regulated in the present study (data not shown), thus clearly arguing that the infection itself and not differences in passage numbers explains the observed transcriptomic differences. Moreover, the fact that PCA identified only one major principal component substantiates that the observed variance in the data sets is indeed attributed to the persistent infection of the cells.

Based on the observation of up‐regulated genes involved in immune processes, and in the light of the fact that HS cells possess properties of macrophages, we investigated whether CDV might exert oncolytic properties through the induction of a polarization in DH82 cells themselves into the tumour‐suppressive M1 or the tumour‐progressive M2 phenotype 14, 18, 41, respectively. Indeed, CDV infection induces an inhibitory phenotype in canine monocyte‐derived dendritic cells *in vitro* 19. Although there were substantial changes in the expression of 40 M1‐ and M2‐related genes (Fig. 3), these genes splitted into a highly comparable number for the M1 and the M2 category, respectively, suggesting that persistent CDV infection is obviously not able to shift the expression pattern towards a distinct polarization phenotype *in vitro*.

Interestingly, reduced angiogenesis has been highlighted previously as a putative oncolytic mechanism for viral oncolysis 42. Solid tumours, with diameters of more than 1–2 mm, require their own blood vessel supply, either by neo‐angiogenesis or vasculogenesis 43. Consequently, tumour progression is associated with an angiogenic switch with a dominance of pro‐angiogenic factors within the tumour microenvironment 44, 45. Strikingly, processes related to angiogenesis were markedly enriched for the down‐regulated genes in DH82‐Ond‐pi. These findings strongly indicate that persistent CDV infection might exert oncolytic properties by inhibiting angiogenesis in HS. The enriched biological term included genes such as *VEGFB* and *thrombospondin 2*, which code for therapeutically relevant proteins implied in tumour angiogenesis 46, 47. In oncolytic virotherapy, the VEGF pathway seems to be a promising target to inhibit tumour growth 48. In contrast to VEGFB, which is controversially discussed in tumour angiogenesis 49, 50, the role of VEGFA as a pro‐angiogenic protein is indisputable 51. With an FC of ‐1.7, *VEGFA* did not reach the FC filtering criteria. Nonetheless, this indicates that CDV infection induces a diminished expression of multiple VEGF pathway members. However, the GO terms related to blood vessel formation also comprised genes, which are known to possess anti‐angiogenic properties such as *thrombospondin 2* 52. This indicates that persistent CDV infection rather affects multiple genes, involved in both angiogenesis and their inhibition.

Based on these observations, tumour vascularization of neoplasms composed of non‐infected DH82 cells or DH82‐Ond‐pi in xenotransplanted mice was determined by immunohistochemistry in an *in vivo* model. In this model, non‐infected transplants showed a significantly higher density of CD31‐positive blood vessels compared to persistently infected transplants at day 7, 14 and 21 after xenotransplantation, associated with a decreased tumour volume in infected transplants. This was paralleled by relatively large areas of necrosis in persistently infected tumour transplants, thus probably representing the result of ischaemic cell death. Moreover, there was a decreased proliferation index in persistently infected tumours, as demonstrated by lower mitotic rate and decreased Ki‐67 immunoreactivity. The results of the xenotransplantation model overall confirmed that persistent infection of DH82 cells with CDV is indeed accompanied by viral oncolysis and that reduced angiogenesis might represent a crucial factor for this mechanism.

Although transcriptome data identified genes, which possess both pro‐ and anti‐angiogenic properties, it appears that the dysregulation of genes involved in blood vessel generation indeed favours reduced angiogenesis *in vivo*. However, whether the observed transcriptomic changes are unique to CDV and a persistent infection state needs to be discussed carefully, as independent persistently infected cells have not been investigated in the present study. However, arguing against the assumption that the persistent nature of the infection represents a prerequisite of CDV‐induced reduced angiogenesis and favouring the hypothesis that similar mechanisms might potentially also account for acute infection models and specifically for CDV, an angiogenesis‐inhibiting effect of CDV has previously been similarly suggested in Vero cells, acutely infected with CDV 53. In this study, CDV triggered a relocation of angiogenesis‐inhibiting vasostatin to the cell surface of infected cells 53.

The provided results have indirect therapeutic relevance, as vaccine strains of CDV are readily available, well tolerated and raise the opportunity of a systemic application in canine patients. Although the approach prevents definitive conclusions on the effects of a more clinically relevant acute infection model of localized HS *in vivo*, we chose a persistently infected cell line with a high number of infected cells to demonstrate virus‐induced differences between infected and non‐infected cells to the highest extend. Additional studies are needed to extend the presented findings on both acute infection models and the systemic form of this malignancy. In particular, it remains to be shown whether the persistent nature of the infection represents a prerequisite for the demonstrated oncolytic mechanisms or whether an acute CDV infection of HS is similarly capable of inducing the observed changes. If an application of attenuated vaccine strains of CDV indeed influences tumour vascularization of HS *in vivo*, we propose that similar mechanisms might also apply to measles virus infection of HS in human beings. The results thus validate the spontaneous canine model as an interesting translational animal model for cancer research, warranting future investigations upon the role of reduced angiogenesis in HS, infected with an oncolytic paramyxovirus.

## Conflict of interest

The authors confirm that there are no conflict of interest.
